# DNA signals at isoform promoters

**DOI:** 10.1038/srep28977

**Published:** 2016-06-29

**Authors:** Zhiming Dai, Yuanyan Xiong, Xianhua Dai

**Affiliations:** 1 School of Data and Computer Science, Sun Yat-Sen University, Guangzhou 510006, China; 2 Guangdong Province Key Laboratory of Big Data Analysis and Processing, Sun Yat-Sen University, Guangzhou 510006, China; 3 Key Laboratory of Gene Engineering of the Ministry of Education and State Key Laboratory of Biocontrol, School of Life Sciences, Sun Yat-Sen University, Guangzhou 510006, China; 4 SYSU-CMU Shunde International Joint Research Institute, Shunde, China; 5 School of Electronics and Information Technology, Sun Yat-Sen University, Guangzhou 510006, China

## Abstract

Transcriptional heterogeneity is extensive in the genome, and most genes express variable transcript isoforms. However, whether variable transcript isoforms of one gene are regulated by common promoter elements remain to be elucidated. Here, we investigated whether isoform promoters of one gene have separated DNA signals for transcription and translation initiation. We found that TATA box and nucleosome-disfavored DNA sequences are prevalent in distinct transcript isoform promoters of one gene. These DNA signals are conserved among species. Transcript isoform has a RNA-determined unstructured region around its start site. We found that these DNA/RNA features facilitate isoform transcription and translation. These results suggest a DNA-encoded mechanism by which transcript isoform is generated.

Promoter DNA sequence features are indicative of gene activity^1^,^2^. DNA consensus sequences in promoters recruit general transcription factors (GTF) and RNA polymerase II (Pol II) to initiate transcription. The sequence context affects selection of transcription start sites (TSS) and transcriptional activity^3^,^4^,^5^. The best-known consensus sequence element is the TATA box, bound by TATA binding protein (TBP)^6^. Experimental evidence indicates that variation of the TATA box sequence results in differences in promoter activity levels^7^,^8^,^9^. DNA sequence also affects chromatin structure, the basic unit of which is the nucleosome^10^,^11^. The DNA-encoded nucleosomal organization in promoter regions plays a role in regulation of gene transcriptional activity^12^,^13^. Rigid DNA enriched of A/T nucleotides is prevalent in upstream regions of TSS, inhibiting DNA packaging of nucleosomes and facilitating the recruitment of Pol II and GTF for transcription^14^,^15^.

The dynamic usage of transcript isoforms is a pervasive mechanism in gene regulation. Its functions have been extensively studied for several individual genes. The diversity in transcript isoforms, mediated through alternate promoter usage and alternative polyadenylation, plays a role in messenger RNA transcription, stability and translation^16^,^17^,^18^,^19^. For example, p53 isoforms with alternative promoters are differentially expressed in a tissue-dependent manner, controlling cell proliferation^20^,^21^. Transcript isoforms are also produced by alternative splicing, encoding proteins that differ in localization or function^22^,^23^. For example, two alternatively spliced variants of TRPM3, which encodes a type of cation-selective channel in human, target different ions^24^. More recent studies have revealed the importance of transcript isoforms in human diseases such as cancer. The M2 isoform of pyruvate kinase promotes cancer metabolism and tumour growth compared with another isoform M1 25.

Human genes have multiple TSSs, which have epigenetic and genomic relevance^26^. However, previous studies have found that most genes have only one major TSS in *Saccharomyces cerevisiae* by RNA sequencing^27^,^28^. We referred to the transcript initiated from the major TSS as main isoform. A recent study has jointly sequenced the 5′ and 3′ ends of each RNA molecule to measure transcript isoforms in *Saccharomyces cerevisiae*^29^. This new sensitive technique revealed that most yeast genes have various TSSs: an average of 26 transcript isoforms covering the intact ORF were expressed per protein-coding gene. Most of these TSSs have not been discovered by RNA sequencing in previous studies^27^,^28^. These newly discovered TSSs are likely to be minor TSSs, that is, the proportion of transcripts initiated from minor TSSs is lower than those initiated from major TSSs. We referred to the transcripts initiated from minor TSSs as other isoforms. It is interesting to examine whether DNA consensus sequences also exist in upstream regions of other isoforms, implying that isoform transcription may be encoded in DNA sequence. If this is true, it is more likely that the extensive isoform diversity has functional relevance. In this study, using genome-wide isoform data in yeast^29^, we revealed DNA signals in upstream regions of other isoforms. These sequence features enhance mRNA and protein expression levels, moreover, are conserved among species.

## Results

### TATA box is enriched in upstream of other isoforms

**Figure 1:**
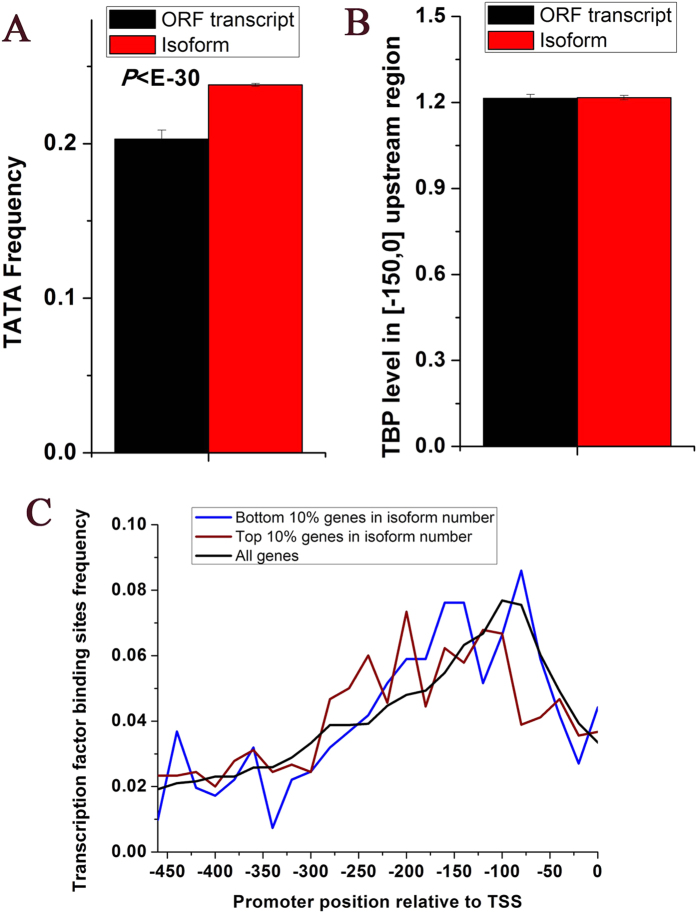
A canonical organization of transcription machinery upstream of other isoforms. (**A**) Average values that correspond to TATA frequency in upstream [−150,0] region are shown for other isoforms (transcript isoforms) (*N* = 162,379) and main isoforms (ORF transcripts) (*N* = 4,759). (**B**) Average values that correspond to PIC occupancy level in upstream [−150,0] region are shown for other isoforms (*N* = 19,808) and main isoforms (*N* = 4,759). (**C**) Distribution of the promoter positions of transcription factor binding sites in the three gene classes: genes with most other isoforms (*N* = 475), genes with least other isoforms (*N* = 475), and all genes (*N* = 4,759). Error bars in (**A**,**B**) were calculated by bootstrapping. The statistical significant values calculated from Mann-Whitney U-test were indicated.

We used genome-wide identified transcript isoforms covering the intact ORF in *S. cerevisiae*^29^. First, we examined the enrichment of TATA box upstream of other isoforms. TATA box generally locates within 150 bp upstream of gene, which recruits pre-initiation complex (PIC) for transcription initiation^6^. For each isoform, we searched the TATA box TATAWAWR consensus^30^ in its 150 bp upstream region. We found that consensus TATA box frequency of other isoforms is significantly higher than that of main isoforms (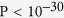, Mann-Whitney U-test, Fig. 1A). This observation might be confused by the shared upstream region between other isoforms and main isoforms. We restricted the analysis to other isoforms whose 150 bp upstream region has no overlap with the 150 bp upstream region of their corresponding main isoforms. Similar result could be observed (Figure S1), albeit with less statistical significance.

Second, we asked whether the observed TATA box is associated with transcription initiation. To this end, we tested whether PIC is enriched in upstream regions of other isoforms. To avoid the shared PIC between other isoforms and main isoforms, we restricted the analysis to other isoforms whose 150 bp upstream region has no overlap with the 150 bp upstream region of their corresponding main isoforms. We found that TBP occupancy in upstream region is comparable between other isoforms and main isoforms (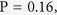 Mann-Whitney U-test, Fig. 1B), indicating that TATA box in upstream region of other isoforms can recruit TBP.

Third, we examined the relationship between other isoforms and transcription factor binding site (TFBS). TFBSs are generally enriched 50–150 bp upstream of the gene in *S. cerevisiae*^15^. However, TFBSs are highly localized in 200–300 bp upstream of greatly other isoform-enriched genes (Fig. 1C). Considering that the start sites of other isoform are upstream of the start codon, this shift in TFBS distribution reflects the role of upstream TFBSs in transcription initiation of other isoforms.

### Other isoforms show low DNA-encoded nucleosome occupancy in upstream regions

**Figure 2:**
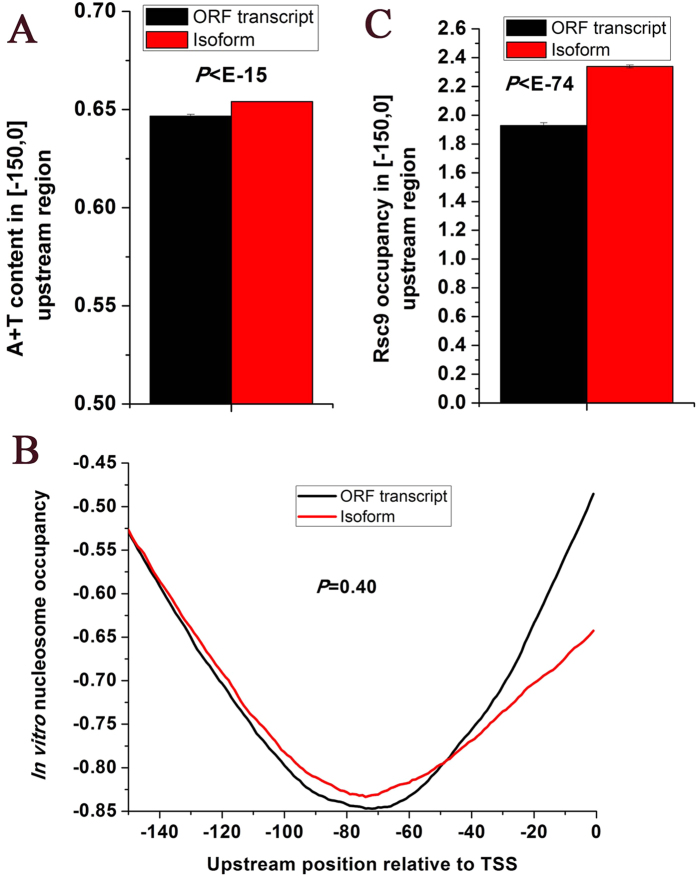
Low DNA-encoded nucleosome occupancy upstream of other isoforms. (**A**) Average values that correspond to A + T content in upstream [−150,0] region are shown for other isoforms (*N* = 19,808) and main isoforms (*N* = 4,759). (**B**) Average *in vitro* nucleosome occupancy profiles within upstream [−150,0] region are shown for other isoforms (*N* = 19,808) and main isoforms (*N* = 4,759). (**C**) Average values that correspond to chromatin remodeler Rsc9 occupancy level in upstream [−150,0] region are shown for other isoforms (*N* = 19,808) and main isoforms (*N* = 4,759). Error bars in (**A**,**C**) were calculated by bootstrapping. The statistical significant values calculated from Mann-Whitney U-test were indicated.

Intrinsic DNA sequence is an important determinant of nucleosome positioning. Nucleosome positioning can be predicted by DNA sequence^31^,^32^. Yeast genes generally contain high A/T content in 150 bp upstream regions^33^,^34^, which inherently inhibit nucleosome formation^35^,^36^. Nucleosome positioning upstream of genes is a barrier for recruitment of GTF and Pol II. Depletion of the nucleosome positioning can enhance transcription. We asked whether other isoforms have similar DNA-encoded nucleosomal organization in their upstream regions. First, we found that other isoforms have higher A/T content in upstream regions than main isoforms (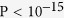, Mann-Whitney U-test, Fig. 2A). Similar observation could be reproduced when using another criterion of calculating A/T content (Figure S2). Second, we examined nucleosome occupancy upstream of other isoforms. Genome-wide occupancy of nucleosomes assembled on purified yeast genomic DNA was measured with 1-bp resolution^14^. This *in vitro* nucleosome map is determined only by the intrinsic sequence preferences of nucleosomes. Other isoforms show comparable *in vitro* nucleosome occupancy in upstream regions with main isoforms (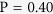, Mann-Whitney U-test, Fig. 2B). Similar results could be reproduced when using another independent *in vitro* nucleosome occupancy data^37^ (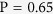, Mann-Whitney U-test). However, other isoforms show higher *in vivo* nucleosome occupancy than main isoforms (Figure S3). Note that experimentally measured nucleosome occupancy *in vivo* is the average among cell populations. Nucleosome positioning might be transiently remodeled to allow the binding of PIC to DNA for transcription initiation. A recent study has found that AT-rich sequences facilitate the remodeling of nucleosomes by the RSC chromatin remodeling complex in gene promoters^38^. Indeed, other isoforms have significantly higher Rsc9 occupancy in upstream regions than main isoforms (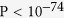, Mann-Whitney U-test, Fig. 2C).

### DNA sequence upstream of other isoforms is conserved among species

**Figure 3:**
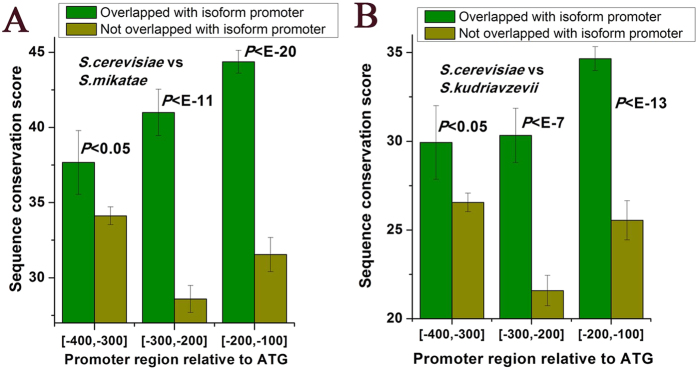
DNA sequence upstream of other isoforms is conserved among species. We performed global alignment on promoter sequences (upstream of start codon) between orthologous genes, and used the resulting alignment score as sequence conservation score. Average values that correspond to sequence conservation score are shown for gene promoters covered by isoform promoters (150 bp upstream) and those not covered by isoform promoters. As [−100, 0] in all gene promoters are covered by isoform promoters, we excluded this region for analysis. Promoter is divided into three bins. (**A**) Orthologous genes between *S. cerevisiae* and *S. mikatae.* (**B**) Orthologous genes between *S. cerevisiae* and *S. kudriavzevii*. Error bars were calculated by bootstrapping. The statistical significant values calculated from Mann-Whitney U-test were indicated.

An interesting question is to ask whether the observed DNA sequence features upstream of other isoforms have roles in regulation. If this is the case, DNA sequence upstream of other isoforms should be under evolutionary constraint to maintain its function. We classified gene promoter regions not covered by isoforms into two catalogues: one covered by 150 bp upstream region of isoform, and the other not. DNA sequence is more conserved among yeast species in former region (Fig. 3). In addition, DNA sequence conservation in gene promoters shows no correlation with transcriptional activity (Figure S4), ruling out the possibility that our observation is biased by transcriptional activity.

### Other isoforms are associated with increased gene expression

**Figure 4:**
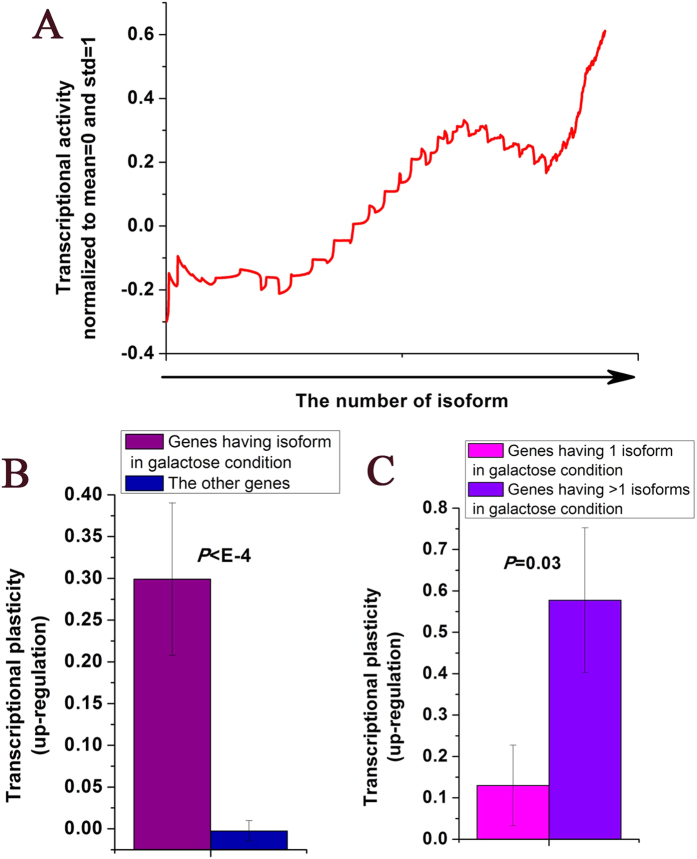
Other isoforms are associated with increased gene expression. (**A**) Genes were ordered by their numbers of other isoforms, and a sliding window (window size of 300 genes) is shown for transcriptional activity, which is normalized by subtracting their means and dividing by their standard deviations. (**B**) Average values that correspond to transcriptional plasticity (up-regulation) are shown for genes having no other isoform in YPD medium but having at least one other isoform in galactose condition, and the other genes. (**C**) Average values that correspond to transcriptional plasticity (up-regulation) are shown for genes having no other isoform in YPD medium but having one other isoform in galactose condition, and genes having no other isoform in YPD medium but having more than one other isoforms in galactose condition. Error bars in (**B**,**C**) were calculated by bootstrapping. The statistical significant values calculated from Mann-Whitney U-test were indicated.

As we have shown that upstream DNA sequence could facilitate transcription of other isoforms, we asked whether genes having other isoforms show high gene expression levels. Indeed, gene transcriptional activity in YPD medium tend to increase with the number of other isoforms (Fig. 4A). Similar results could be reproduced when using another independent transcriptional activity data^28^ (Figure S5). We next tested whether this relationship could be observed in other cellular conditions. We identified genes having no other isoform in YPD medium but having other isoform when the yeast was grown in a galactose medium. These genes show higher degree of transcriptional up-regulation in various cellular conditions relative to YPD medium (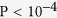, Mann-Whitney U-test, Fig. 4B). Moreover, high degree of up-regulation is associated with high number of other isoforms (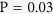, Mann-Whitney U-test, Fig. 4C). Similar results could be reproduced when using another independent transcriptional plasticity data^39^ (Figure S6). A previous study has found that genes with high upstream nucleosome occupancy close to the start codon show higher degree of transcriptional up-regulation in various cellular conditions, and referred these genes as occupied proximal-nucleosome (OPN) genes^40^. The genes (*N* = 199) we identified above, which have no other isoform in YPD medium but have at least one other isoform in galactose condition, only show small overlap (12 genes) with OPN genes (*N* = 544) (hypergeometric 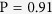). Moreover, these genes show comparable degree of transcriptional up-regulation with OPN genes (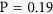, Mann-Whitney U-test, Figure S7). These results suggest that genes with other isoforms are up-regulated in a different mechanism from OPN genes.

### Other isoforms are associated with increased translation efficiency

**Figure 5:**
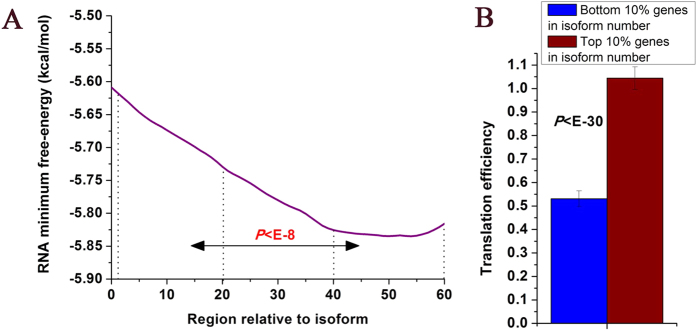
Other isoforms are associated with increased translation efficiency. (**A**) Average RNA minimum free-energy profile computed by RNA sequence alone is shown for other isoforms. The profile is smooth by a 15 bp sliding window. The statistical significant difference between [0,20] and [40,60] regions calculated from Mann-Whitney U-test were indicated. (**B**) Average values that correspond to translation efficiency are shown for genes with most other isoform and genes with least other isoform. Error bars were calculated by bootstrapping. The statistical significant values calculated from Mann-Whitney U-test were indicated.

RNA structure has critical roles in regulation of translation. mRNA generally has unstructured (accessible) region immediately upstream of the start codon, which might facilitate ribosome binding and translation initiation^41^,^42^. We asked whether other isoforms also have unstructured region near their start sites. As experimentally measured RNA structure data is not available for other isoforms, we instead used RNA free-energy calculated solely by RNA sequence. RNA free-energy is negatively correlated with RNA structure: High RNA free-energy corresponds to an RNA unstructured region. RNA encodes relatively unstructured region near start sites of other isoforms (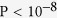, Mann-Whitney U-test, Fig. 5A). We tested whether this RNA structure has consequent effects on translation. Indeed, using genome-wide ribosome profiling data^43^, we found that genes having more other isoforms show higher translation efficiency (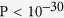, Mann-Whitney U-test, Figs. 5B and S8 and S9). As long mRNAs can have more ribosomes than short mRNAs, we examined whether our result is biased by this property. However, we found that genes having more other isoforms have shorter mRNA (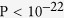, Mann-Whitney U-test, Figure S10).

## Discussion

In this study, we performed a genome-wide analysis and investigated into the cause and consequence of transcript isoforms. We found that a gene’s main isoform and other isoforms, produced from alternative transcriptional start sites, show similar patterns within their DNA promoter sequences. In particular, we found TATA box features, nucleosome-disfavored sequence signals, and DNA-encoded RNA unstructured regions near the respective transcriptional start sites. These patterns facilitate isoform transcription and translation. Main results in this study could be reproduced using another independent TSS data^28^ (Figures S11–S13). These results indicate that prevalent other isoforms are encoded in DNA sequence, and have implications in isoform function inference. As function of most transcript isoforms is unknown, it is interesting to examine whether isoform function can be inferred from its DNA context.

One key finding in this study is that DNA sequence upstream of other isoforms is conserved among species. DNA signal facilitating isoform transcription is under selective constraint. Transcription initiation includes two steps: chromatin remodeling and PIC assembly. Although there is no low nucleosome occupancy upstream of isoform, the intrinsic nucleosome-disfavored DNA signals upstream of isoform could facilitate nucleosome repositioning by chromatin remodelers. TATA box upstream of isoform recruit PIC for transcription initiation.

Genes with more other isoforms have more TSSs (Figure S14). Multiple TSSs upstream of ORF enhances the chance of transcription initiation, thereby genes with more other isoforms show high transcriptional activity. Some genes utilize this strategy in response of environmental condition changes. To the best of our knowledge, this is a new strategy ever revealed. As we have shown that isoform generation is encoded in DNA sequence, their response of environmental condition changes might be also encoded in DNA sequence. Further experiments will be needed to examine why these genes have no other isoform in normal condition.

## Methods

### Data preparation

Yeast genome-wide transcript isoform coordinate data in YPD medium were taken from Pelechano *et al*.^29^. Genome-wide occupancy data for PIC (including TBP, TFIIA, TFIIB, TFIID, TFIIE, TFIIF, TFIIH and TFIIK) were taken from Rhee *et al*.^30^. Average PIC occupancy level in upstream [−150,0] region is calculated for each isoform. Genome-wide binding data corresponding to 203 TFs were taken from MacIsaac *et al*.^44^. A *P* value cutoff of 0.001 was used to define the set of genes bound by a particular TF. Genome-wide nucleosome occupancy data *in vivo* and *in vitro* measured with high resolution were taken from Kaplan *et al*.^14^. Average nucleosome occupancy level in upstream [−150,0] region is calculated for each isoform. Genome-wide chromatin remodeler Rsc9 occupancy data measured with high resolution were taken from Venters *et al*.^45^. Average Rsc9 occupancy level in upstream [−150,0] region is calculated for each isoform. The list of OPN genes were taken from Tirosh *et al*.^40^. Genome-wide gene transcriptional activity (transcription rate) data were taken from Holstege *et al*.^46^. Genome-wide gene translation efficiency (ribosome/mRNA) data were taken from Artieri *et al*.^43^. All these data in form of processed data were downloaded from supplemental materials or supplemental websites of their original literatures. All data in this study were measured in YPD medium unless indicated.

### Definition of other isoform

1) We restricted the analysis to isoforms covering the whole ORF. In this way, these isofroms are transcripts of ORFs, and are not noise of transcription. ORF and their coordinate (start and stop codons) data were taken from *Saccharomyces* Genome Database^47^. 2) We excluded isoforms whose coordinates are the same as major TSS of their covering ORF. In this way, we can separate isoform transcripts initiated from minor TSSs (other isoform) from isoform transcripts initiated from major TSSs (main isoform). Experimentally validated TSS data for ORF were taken from Miura *et al*.^27^. In this study, we used isoforms identified by 1) and 2) as other isoforms and compared them with main isoforms (i.e. ORF transcripts in this study, Figure S15).

### Calculation of promoter sequence conservation

We classified gene promoter regions not covered by isoforms into two catalogues: one covered by 150 bp upstream region of isoforms, and the other not. Orthologous genes between *S. cerevisiae* and *S. mikatae*, between *S. cerevisiae* and *S. kudriavzevii* were taken from Wapinski *et al*.^48^. We performed global alignment (function ‘nwalign’ in software ‘Matlab’ version R2012b) on promoter sequences between orthologous genes, and used the resulting alignment score as sequence conservation score. We then used these scores to compare between the two promoter catalogues identified above.

### Calculation of transcriptional plasticity

We compiled available gene expression data from the Stanford Microarray Database^49^, a total of 1,260 published microarray experiments for 6,260 genes in various cellular conditions. For each gene, we calculated the average of the squared positive expression level from the 1,260 experiments, and defined the normalized resulting value as transcriptional plasticity (up-regulation), which reflected the dynamic extent of its expression level in various conditions.

### Calculation of RNA free energy

The minimum free energy for the RNA sequence was computed as previous studies^50^,^51^. For each of sliding windows (50 bp long, 1 bp step) within the transcript isoform, we computed the minimum free energy (function ‘rnafold’ in software ‘Matlab’ version R2012b) in that window, and assigned it to the first nucleotide of the window.

### Statistical methods

Given two samples of values, the Mann-Whitney U-test (function ‘ranksum’ in software ‘Matlab’ version R2012b) is designed to examine whether they have equal medians. The main advantage of this test is that it makes no assumption that the samples are from normal distributions. Error bars in figures were calculated by bootstrapping: Data points in a data set are randomly resampled to create 1000 different data sets (each has the same number of data points as the original data set, function ‘bootstrp’ in software ‘Matlab’ version R2012b), and the mean value is computed for each data set, and standard deviation is computed for the 1000 mean values.

## Additional Information

**How to cite this article**: Dai, Z. *et al*. DNA signals at isoform promoters. *Sci. Rep.* **6**, 28977; doi: 10.1038/srep28977 (2016).

## Supplementary Material

Supplementary Information
