# Supplementary material for: DNA signals at isoform promoters
Source: Sci Rep. 2016 Jun 29;6:28977. doi: 10.1038/srep28977 (PMC4926256; doi:10.1038/srep28977)
Supplement: Supplementary Information [file srep28977-s1.pdf]

Supplemental Materials

DNA signals at isoform promoters

Zhiming Dai[1,2], Yuanyan Xiong[3,4§], Xianhua Dai[5]

[1]School of Data and Computer Science, Sun Yat-Sen University, Guangzhou 510006, China
[2]Guangdong Province Key Laboratory of Big Data Analysis and Processing, Sun Yat-Sen University, Guangzhou 510006, China
[3]Key Laboratory of Gene Engineering of the Ministry of Education and State Key Laboratory of Biocontrol, School of Life Sciences, Sun Yat-Sen University, Guangzhou 510006, China
[4]SYSU-CMU Shunde International Joint Research Institute, Shunde, China
[5]School of Electronics and Information Technology, Sun Yat-Sen University, Guangzhou 510006, China

Figures. S1 to S15

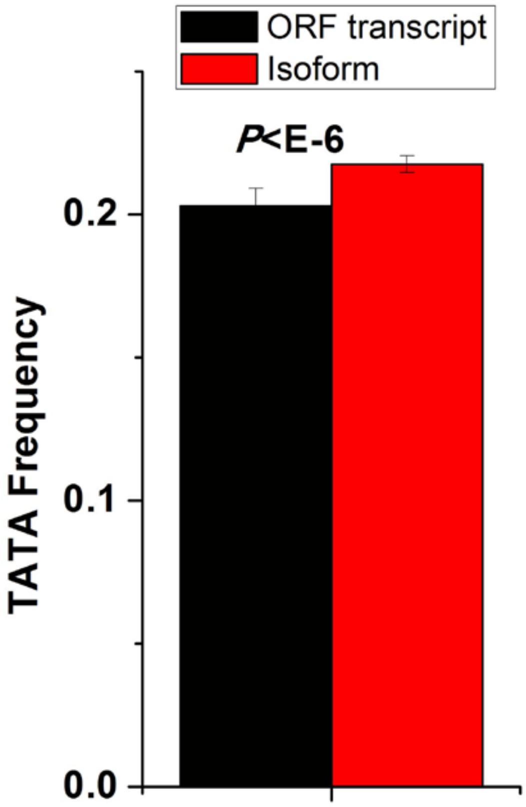

Figure S1. Same as Figure 1A, but restricting the analysis to other isoforms (*N*=19,808) whose 150 bp upstream region has no overlap with the 150 bp upstream region of their corresponding main isoforms.

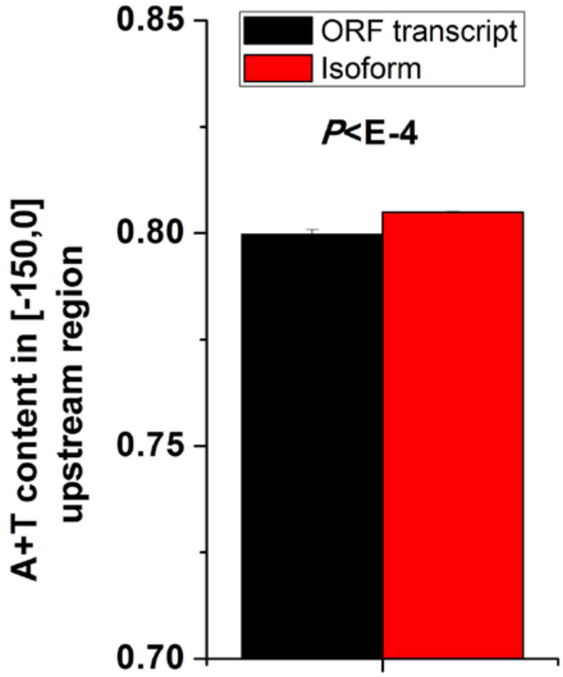

Figure S2. Same as Figure 2A, but for the criterion of calculating A/T content. For each of sliding windows (20 bp long, 10 bp step) within the [-150,0] region, we computed per promoter, the A/T content in that window, and took the maximum over these windows to be A+T content.

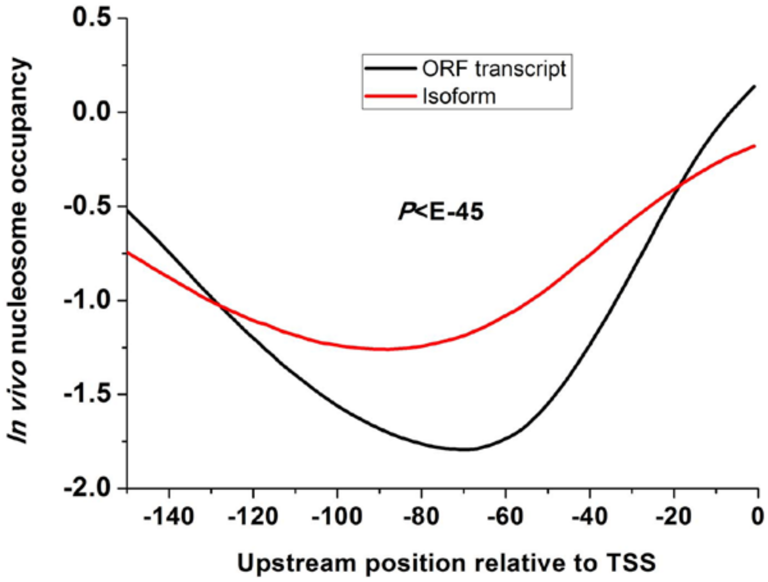

Figure S3. Average *in vivo* nucleosome occupancy profiles within upstream [-150,0] region are shown for other isoforms and main isoforms.

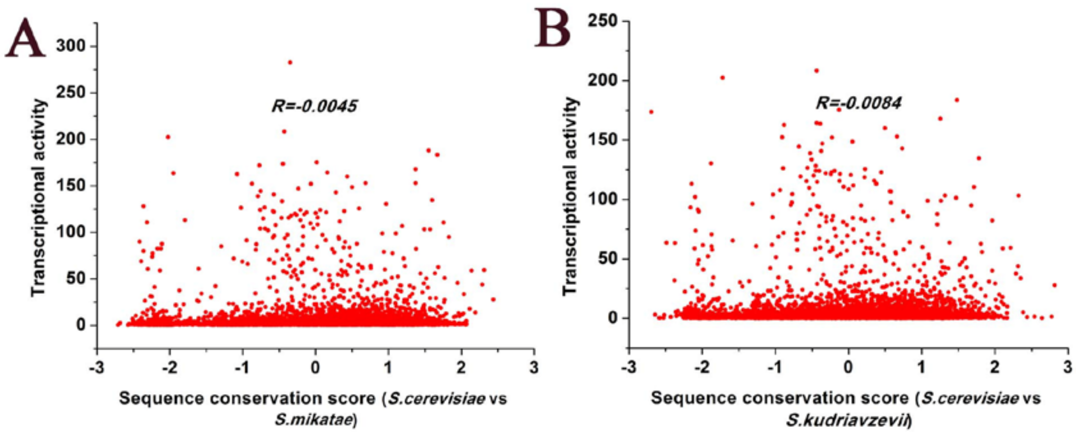

Figure S4. DNA sequence conservation in gene promoters shows no correlation with transcriptional activity. (A) Shown is a scatter plot comparison between sequence conservation score (*S. cerevisiae* and *S. mikatae*) and transcriptional activity. We performed global alignment on promoter sequences (400bp upstream of start codon) between orthologous genes, and used the resulting alignment score as sequence conservation score. The Pearson correlation is indicated. (B) Same as (A), but for orthologous genes between *S. cerevisiae* and *S. kudriavzevii*.

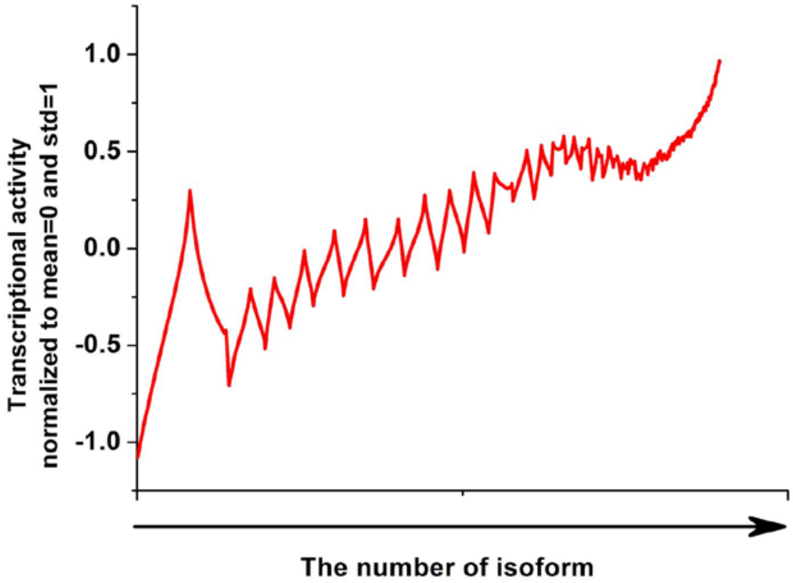

Figure S5. Same as Figure 4A, but using another independent transcriptional activity data[1].

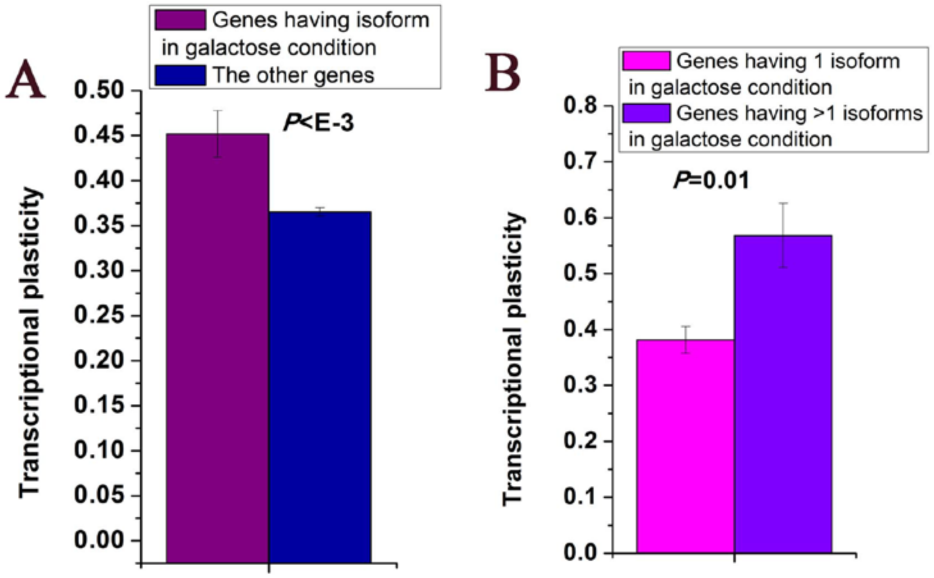

Figure S6. Same as Figure 4B and 4C, but using another independent transcriptional plasticity data[2].

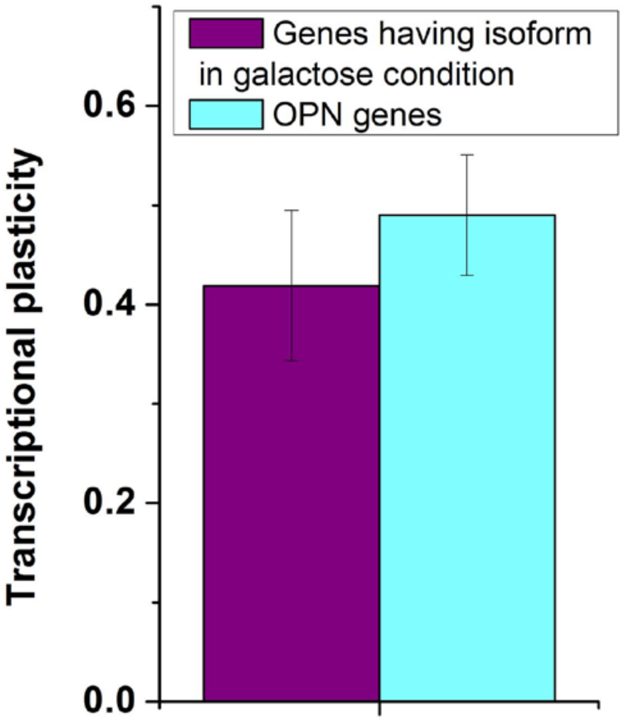

Figure S7. Average values that correspond to transcriptional plasticity are shown for genes having no other isoform in YPD medium but having at least one other isoform in galactose condition, and OPN genes ($P = 0.19$, Mann-Whitney U-test). Error bars were calculated by bootstrapping.

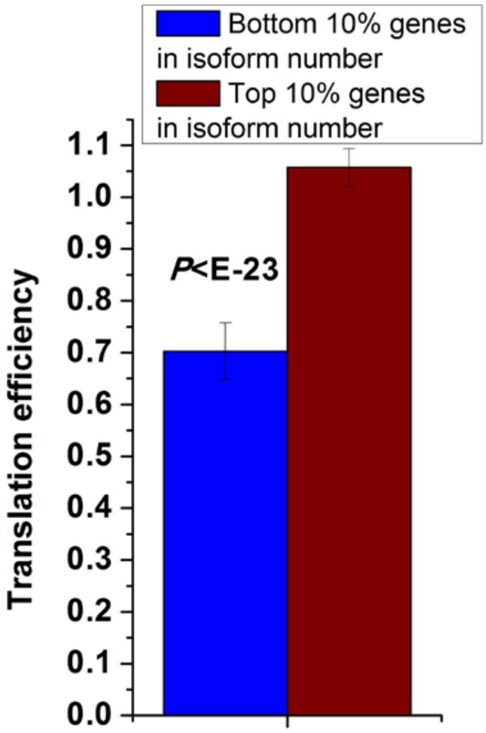

Figure S8. Same as Figure 5B, but using another independent translation efficiency data[3].

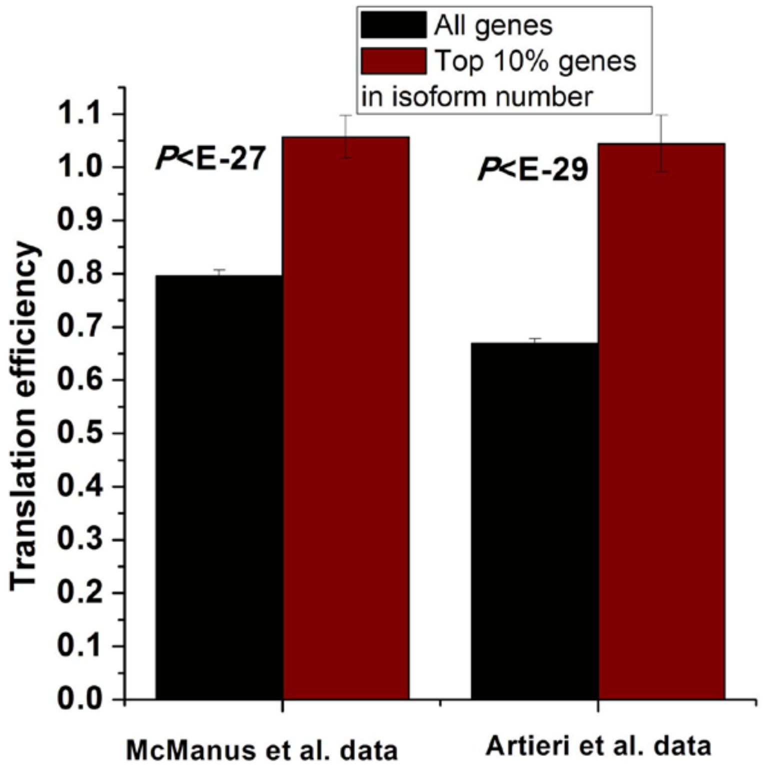

Figure S9. Same as Figure 5B, but for genes with most other isoforms and genes with least other isoform and all genes, by two independent translation efficiency data[3,4].

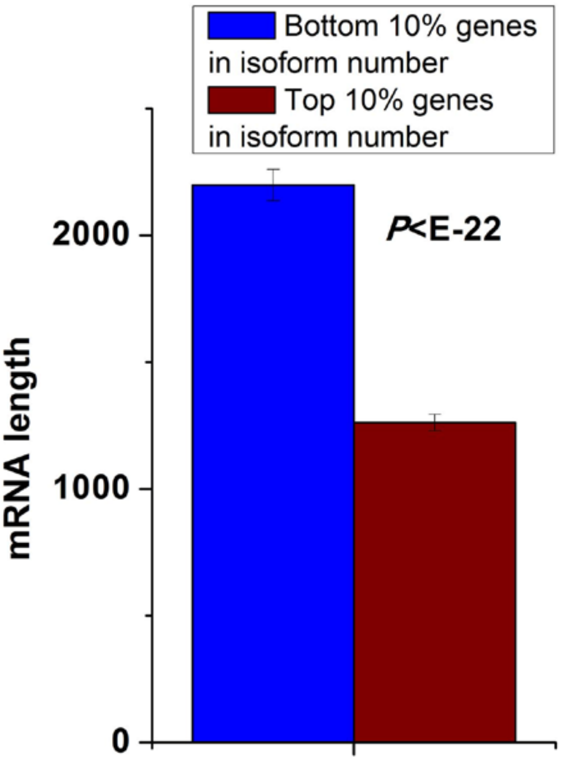

Figure S10. Average values that correspond to mRNA length are shown for genes with most other isoforms and genes with least other isoforms. Error bars were calculated by bootstrapping. The statistical significant values calculated from Mann-Whitney U-test were indicated.

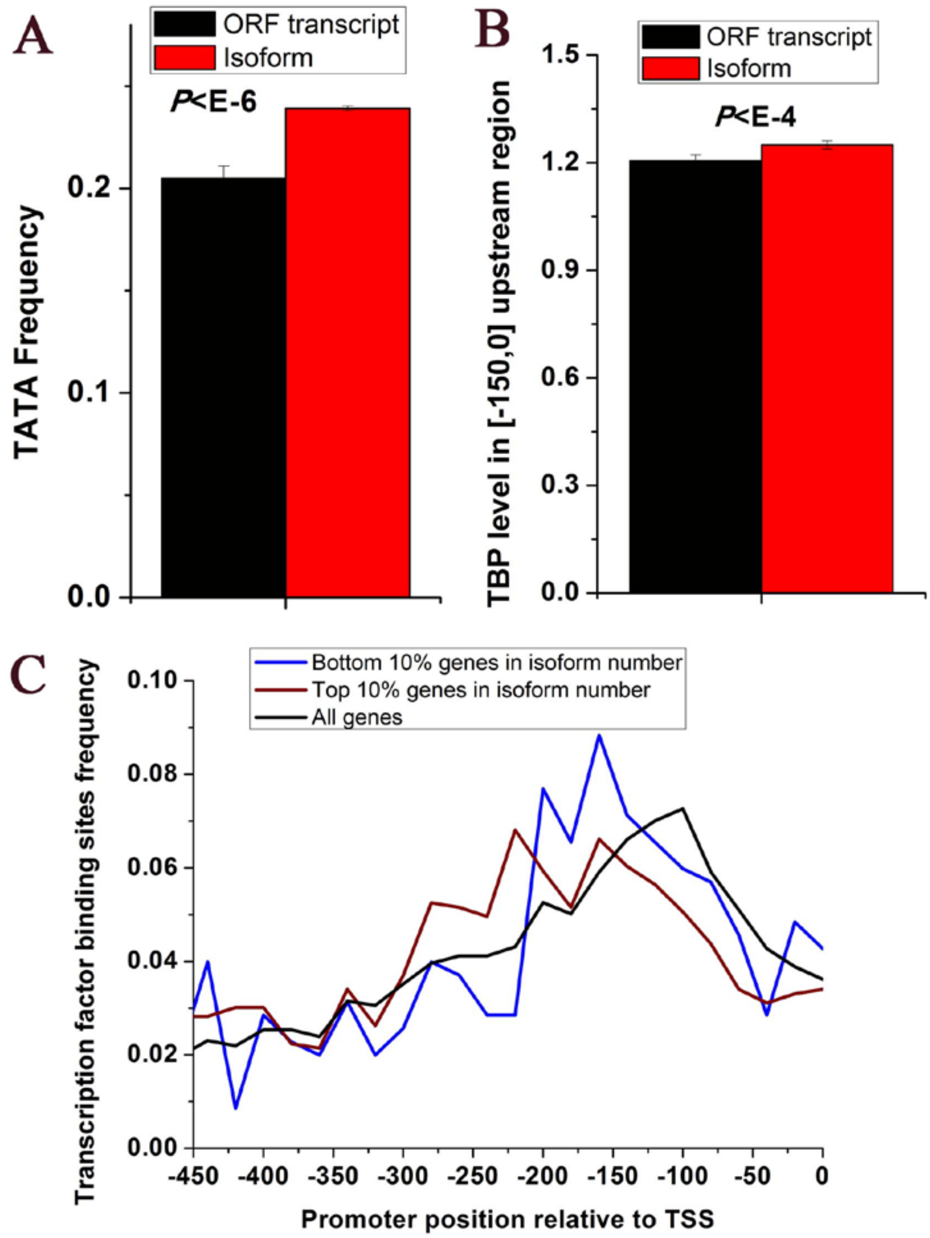

Figure S11. Same as Figure 1, but using another independent TSS data[1].

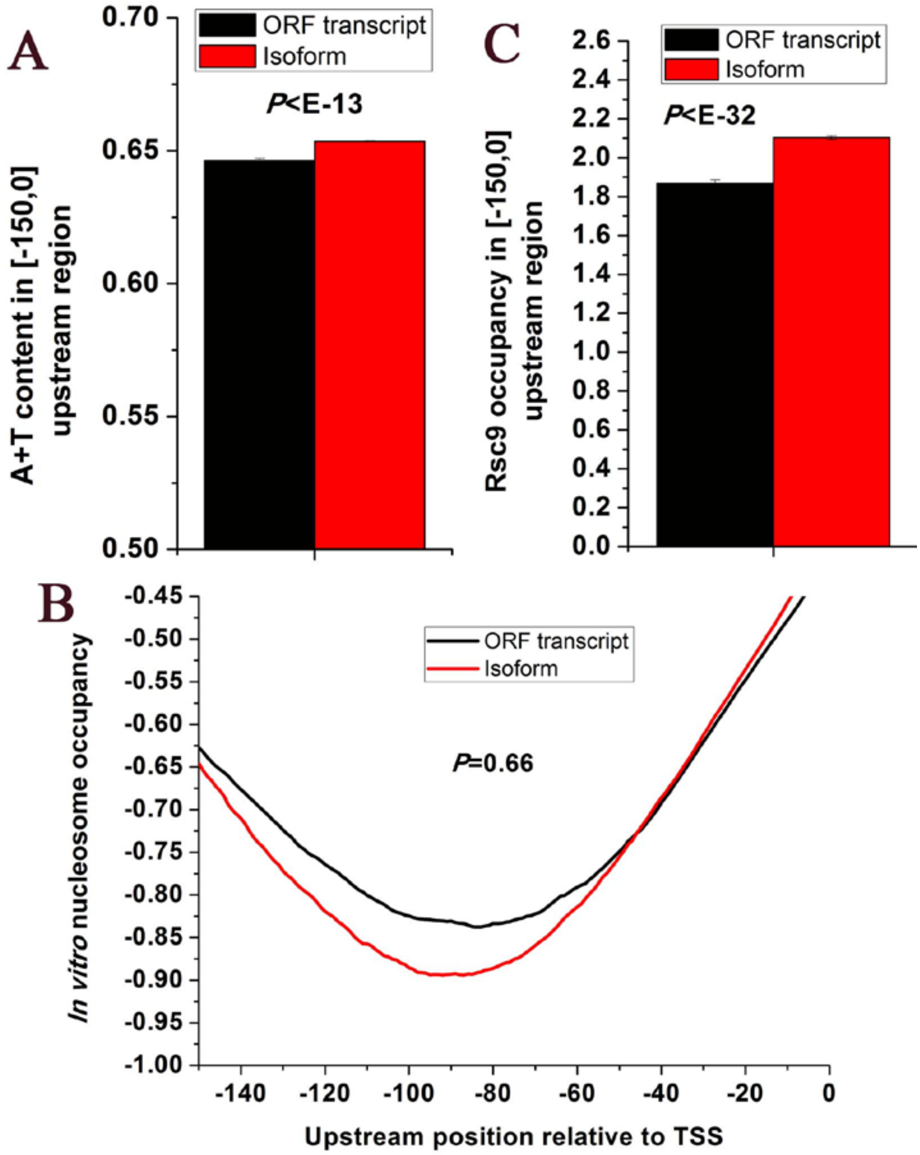

Figure S12. Same as Figure 2, but using another independent TSS data[1].

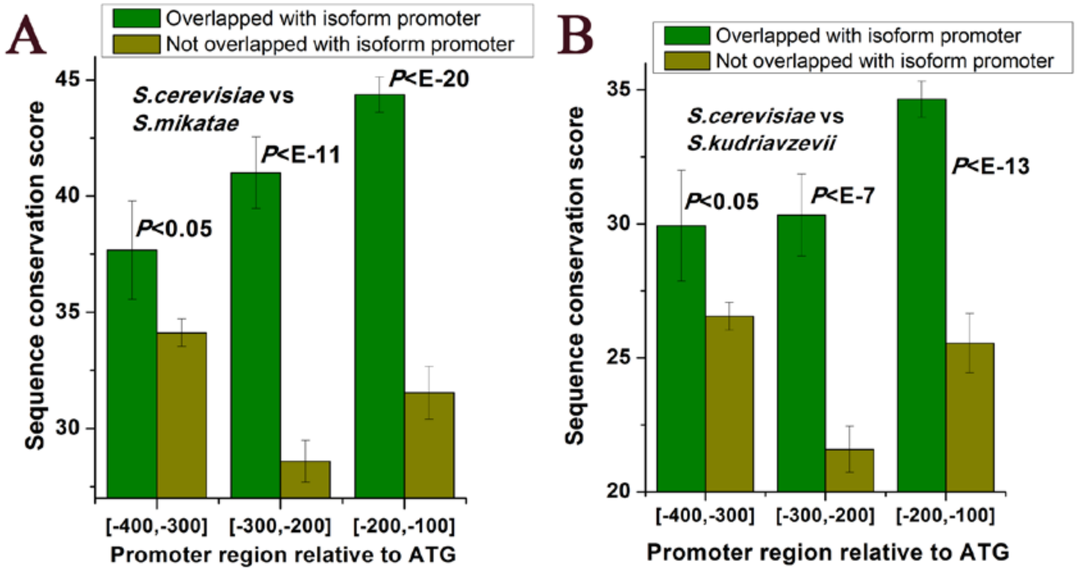

Figure S13. Same as Figure 3, but using another independent TSS data[1].

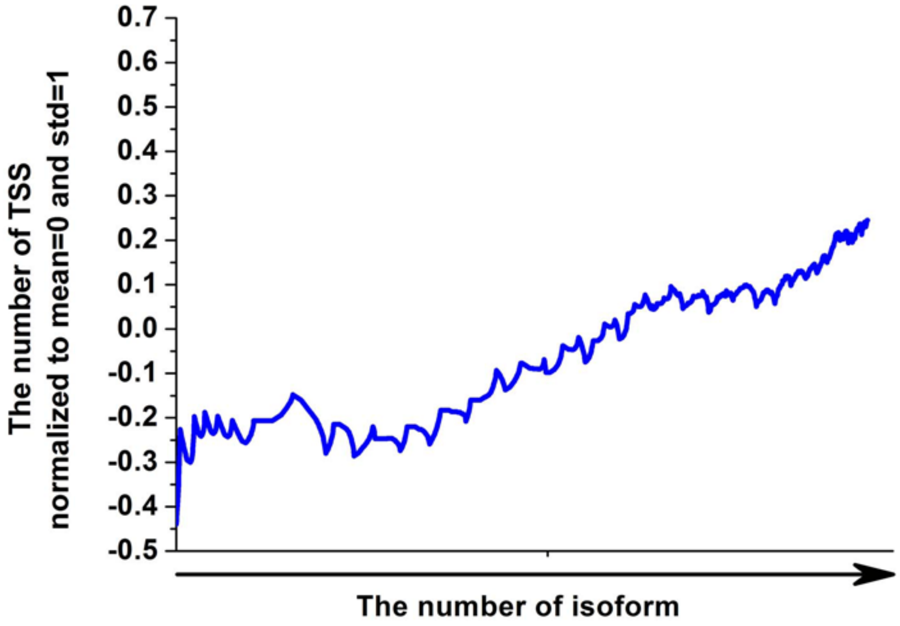

Figure S14. Genes were ordered by their numbers of other isoforms, and a sliding window (window size of 300 genes) is shown for the number of TSSs, which is normalized by subtracting their means and dividing by their standard deviations.

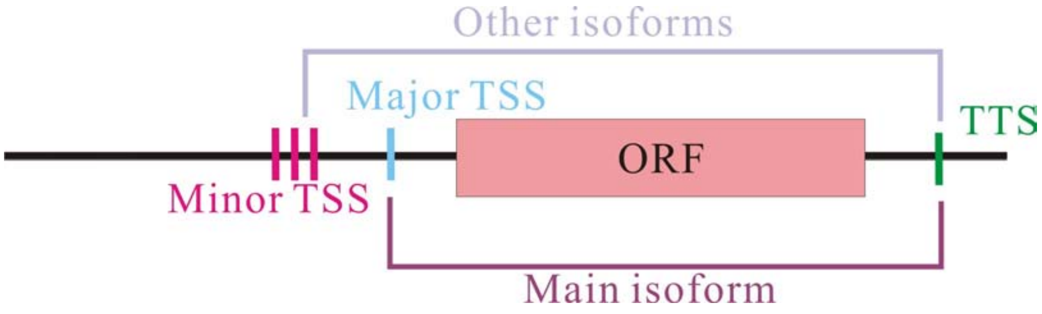

Figure S15. Definition of main isoform (i.e. ORF transcripts) and other isoforms.

References

1 Nagalakshmi, U. *et al.* The transcriptional landscape of the yeast genome defined by RNA sequencing. *Science* **320**, 1344-1349, doi:10.1126/science.1158441 (2008).

2 Lin, Z., Wu, W. S., Liang, H., Woo, Y. & Li, W. H. The spatial distribution of cis regulatory elements in yeast promoters and its implications for transcriptional regulation. *BMC Genomics* **11**, 581, doi:10.1186/1471-2164-11-581 (2010).

3 McManus, C. J., May, G. E., Spealman, P. & Shteyman, A. Ribosome profiling reveals post-transcriptional buffering of divergent gene expression in yeast. *Genome Res* **24**, 422-430, doi:10.1101/gr.164996.113 (2014).

4 Artieri, C. G. & Fraser, H. B. Evolution at two levels of gene expression in yeast. *Genome Res*

24, 411-421, doi:10.1101/gr.165522.113 (2014).
